# Prulifloxacin Effectiveness in Moderate-to-Severe Acute Exacerbations of Chronic Bronchitis: Α Noninterventional, Multicentre, Prospective Study in Real-Life Clinical Practice—The “AIOLOS” Study

**DOI:** 10.1155/2021/6620585

**Published:** 2021-05-25

**Authors:** Konstantinos Gourgoulianis, Alessandro Ruggieri, Alessandra del Vecchio, Fabrizio Calisti, Alessandro Comandini, Giovanna Esposito, Giorgio Di Loreto, Nikolaos Tzanakis

**Affiliations:** ^1^University Respiratory Clinic, University General Hospital of Larissa, Larissa 41110, Greece; ^2^Αngelini Pharma S.p.A., Viale Amelia 70, 00181 Rome, Italy; ^3^University Respiratory Clinic, University General Hospital of Heraklion (PE.PA.GNI), Heraklion 71110, Greece

## Abstract

Real-world evidence regarding the effectiveness of prulifloxacin in the treatment of acute exacerbations of chronic bronchitis (AECB) is limited. Therefore, this study aimed to assess the rates and time to symptom improvement and resolution in patients with moderate-to-severe AECB who were given prulifloxacin in the routine care in Greece. This observational, prospective study, conducted in 15 hospital-based clinics across Greece, enrolled outpatients >40 years old, with moderate-to-severe AECB, for whom the physician had decided to initiate treatment with prulifloxacin. Data were collected at prulifloxacin onset (baseline), 7–10 days after baseline, and at least 28 days after therapy completion. Between 23 November 2015 and 27 January 2018, 305 patients (males: 76.4%; mean (standard deviation) (SD) age: 69.7 (9.8) years; Anthonisen type I/II: 94.8%; chronic bronchitis duration >10 years: 24.9%) were consecutively enrolled. At baseline, >80% had increased sputum volume, cough, dyspnoea, and sputum purulence. Prulifloxacin improved symptoms in 99.7% of the patients after a mean (SD) of 5.47 (3.57) days, while symptoms fully recovered after a mean (SD) of 10.22 (5.00) days in 95.4%. The rate of adverse events related to prulifloxacin was 1.3% (serious: 0.7%). In the routine care in Greece, prulifloxacin was highly effective in moderate-to-severe AECB, while displaying a predictable safety profile.

## 1. Introduction

Chronic bronchitis is a progressive disease defined by at least 3 months of sputum and cough during at least two consecutive years, not attributed to other causes [1, 2]. Recurrent attacks of bronchial inflammation, known as acute exacerbations of chronic bronchitis (AECB), characterized by increased cough, worsening dyspnoea, and changes in sputum purulence and volume, occur 1.5 to 3 times a year and pose a substantial burden to patients, contributing not only to a decline in lung function and impaired quality of life (QoL), but also to increased mortality [1, 3–6].

Infectious agents account for about 80% of AECB episodes, with 50–70% of them being attributed to bacterial causes [1, 2]. The cardinal symptoms of increased sputum volume, sputum purulence, and dyspnoea are used to classify the clinical severity of AECB [7]. According to the Anthonisen criteria used for evaluation of antibiotic therapy use during an exacerbation, patients with all three of these symptoms are classified as type I, those with two as type II, and those with just one of these symptoms and at least one other minor symptom (upper respiratory infection within the past 5 days, fever with no other cause, increased wheezing, increased cough, or increased respiratory or heart rate by 20% compared to baseline) as type III [7]. Patients with type I or II AECB are considered to be those that benefit the most from antibacterial treatment [7].

Among antibiotics used for treating AECBs, fluoroquinolones are considered an advantageous choice compared to other antibiotics, such as macrolides, in terms of higher microbiological success and lower recurrence rates and to amoxicillin/clavulanate, as displaying a better safety profile [8, 9]. A review from the European Medicines Agency (EMA) finalized in November 2018, at which time enrolment in the present study had been completed, on serious, disabling, and potentially permanent side effects associated with fluoroquinolones resulted in a restriction of their use for several indications, including AECB. Specifically, EMA concluded that fluoroquinolones should be used for the management of AECB only when it is considered inappropriate to use other antibacterial agents that are commonly recommended for the treatment of these infections, elaborating that after the review of the new risk data the benefit : risk balance remained unchanged for severe AECB [10].

Prulifloxacin is a broad-spectrum oral fluoroquinolone with activity against various Gram-negative and Gram-positive bacteria commonly associated with lower respiratory tract infections [11, 12]. In patients with AECB, the treatment success rates in randomized clinical trials (RCTs) ranged between 85 and 95% [12–14]. Unfortunately, there is limited information on its real-world effectiveness and its impact on patient-reported outcomes (PROs), including health, overall well-being, and daily life.

In light of the above, this current noninterventional study aimed to assess the effectiveness of prulifloxacin and to examine its effect on the patients' QoL, work productivity, and activity impairment in a representative sample of outpatients with moderate-to-severe AECB treated in routine practice in Greece.

## 2. Materials and Methods

### 2.1. Study Design, Setting, and Population

This was a single-country, multicentre, observational, prospective study conducted in 15 private and public hospital-based respiratory clinics in Greece distributed in seven of the 13 administrative geographic regions of the country. Of the participating study sites, 13 enrolled patients in the study.

Consecutive sampling was utilized as means to control for patient selection bias. Eligible patients were male and female outpatients, who were >40 years old, diagnosed with moderate-to-severe AECB, and prescribed prulifloxacin at 600 mg daily up to 10 days according to the locally approved summary of product characteristics. The physician's decision to prescribe prulifloxacin was made in the context of standard clinical practice, with all precautions relating to the use of the drug, and preceded patient's enrolment, being clearly separated from his/her decision to include the patient in the study. Special warnings listed in the summary of product characteristics include but are not limited to the predisposition of patients with latent or known deficiencies for the glucose-6-phosphate dehydrogenase activity for haemolytic reactions upon treatment with quinolones. Moreover, concomitant treatment with cimetidine, antiacids containing Al and Mg, or preparations containing iron and calcium, as well as concomitant ingestion of milk, reduce the absorption of prulifloxacin; therefore, the timing of administration of the drug relative to the aforementioned agents was adjusted accordingly [15]. Patients were excluded if they had received any investigational product within 1 month or 5 half-lives of the investigational agent (whichever was longer) before commencement of therapy with prulifloxacin.

The observational period for each patient started at the initiation of treatment with prulifloxacin. Baseline was defined as the time of prulifloxacin treatment onset. Data were collected using electronic case report forms at three visits: enrolment [baseline; visit 1], 7–10 days after baseline (visit 2), and at least 28 days after the end of therapy (visit 3), as per standard practice.

The study was designed and performed according to international guidelines, applicable national regulations, and the ethical principles laid down in the Declaration of Helsinki and were approved by the hospitals' Institutional Review Boards. All patients provided written informed consent prior to study participation.

### 2.2. Data Collection

Data were collected by physicians as generated in routine clinical practice and by patients using PROs that comprised the Greek version of the validated Work Productivity and Activity Impairment: Respiratory Symptoms (WPAI : RS) and the EuroQol 5-dimensions 3-levels (EQ-5D-3L) instruments. PROs were optionally completed by the patients at each of the three visits. Baseline data collected included sociodemographic characteristics, classification of the exacerbation according to the Anthonisen criteria [7], history of exacerbations in the last year, spirometry evaluation, and treatments received in the month prior to baseline. Physicians' assessments included clinical symptoms' evaluation at all visits, symptoms' improvement and recovery at visits 2 and 3, and prulifloxacin's overall effectiveness and tolerability. Therefore, the recorded data represent a snapshot of the patient's symptoms at the time of the visit. Information regarding treatment with prulifloxacin and concomitant medications was recorded throughout study participation. Finally, patients were instructed by physicians to report adverse events (AE) continuously and in real time.

### 2.3. Study Objectives and Outcomes

The primary objective was to determine the time until symptoms' improvement, defined as the number of days until improvement of the symptoms of the acute exacerbation, as assessed by the physician. Secondary outcomes included the frequency of cure, expressed as the proportion of patients without symptoms; the course of symptoms, assessed as the proportion of patients experiencing a change in severity of symptoms (graded as absent, mild, moderate, or severe) from the baseline to each postbaseline visit, described as “relieved” (i.e., switched to “absent” at follow-up), “improved” (i.e., switched to a lower severity category at follow-up), “unchanged” (i.e., remaining in the same severity category at follow-up), or “worsened” (i.e., switched to a higher severity category at follow-up) compared to the start of therapy; and prulifloxacin's effectiveness and tolerability as evaluated by the physician on a 4-point scale (“very good,” “good,” “sufficient,” and “insufficient”) and as assessed by the patient by answering the question “Did prulifloxacin help?”, similar to previous studies [16]. Additionally, the effects of prulifloxacin on the patients' QoL and work productivity/activity impairment were assessed using the EQ-5D-3L and WPAI : RS questionnaires, respectively.

### 2.4. Statistical Analysis

Effectiveness outcomes were evaluated in the effectiveness population, that is, all patients who received at least one prulifloxacin dose and had evaluable effectiveness information. Demographic and baseline characteristics and safety analysis were performed in the safety population, that is, all patients who received at least one dose of prulifloxacin. Descriptive statistics are provided as mean, standard deviation (SD) for continuous variables or as absolute and relative frequencies for categorical variables. All statistical analyses were performed using SAS version 9.4.

For sample size justification, assuming a standard deviation of 1.8 days for the mean time to improvement [16], a sample size of 300 patients was planned to estimate the two-sided 95% confidence interval (CI) with a precision of 0.204.

## 3. Results

### 3.1. Patient Disposition

**Figure 1:**
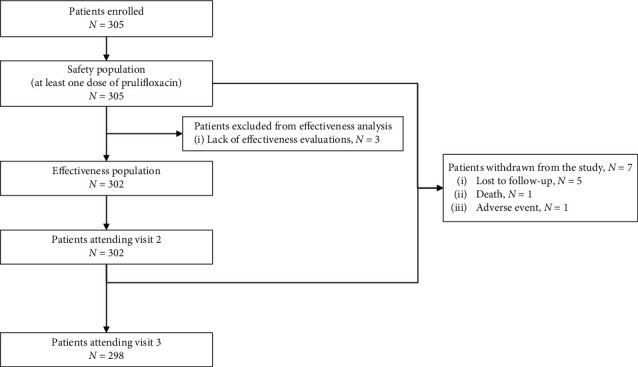
Patient disposition.

Between 23 November 2015 and 27 January 2018, 305 patients were enrolled in the study by 13 hospital clinics throughout Greece. Seven patients were prematurely withdrawn, leading to 298 patients attending visit 3 (Figure 1).

### 3.2. Patient, Disease, and Prior/Concomitant Treatment Characteristics

Most patients were males (76.4%), ≥65 years old (71.1%) with moderate-to-severe airflow obstruction based on the spirometric evaluation (93.8% patients with forced expiratory volume in one second (FEV1) ≤80%) at baseline, at which time patients were under exacerbation. According to Anthonisen criteria, 51.1% of the patients were classified as having type I and 43.6% type II AECB. The mean (SD) bronchitis duration was 8.0 (6.2) years. In the 12 months before enrolment, 77.7% of the patients had experienced at least one exacerbation leading to hospitalisation in 14.8% (Table 1). Based on patients' medical history at baseline, six conditions were listed under “respiratory, thoracic, and mediastinal disorders,” namely, bronchiectasis, dyspnoea, pulmonary embolism, respiratory failure, scoliosis, and sleep apnoea syndrome, each reported for one patient.

Overall, during the last month prior to enrolment, 7.9% of the patients had received drugs for obstructive airway diseases, 2.0% had received corticosteroids for systemic use, and 1.0% (3 patients) had used an antibiotic (clarithromycin or amoxicillin/clavulanate) for the treatment of respiratory tract infections. During study participation, 92.5% of the patients received at least one concomitant medication, with 82.0% receiving drugs for obstructive airway diseases and 18.4% corticosteroids for systemic use. Moreover, four patients received a concomitant antibacterial drug for chronic obstructive pulmonary disease (COPD) exacerbation (azithromycin or amoxicillin with/without clavulanate).

### 3.3. Effectiveness Outcomes: Effects of Prulifloxacin on Chronic Bronchitis Symptoms

Among patients of the effectiveness population, the mean (SD) duration of prulifloxacin treatment was 9.53 (1.28) days (range: 2–10 days), with prulifloxacin administered at the recommended dose in 98.3% of the patients (Table 2).

**Figure 2:**
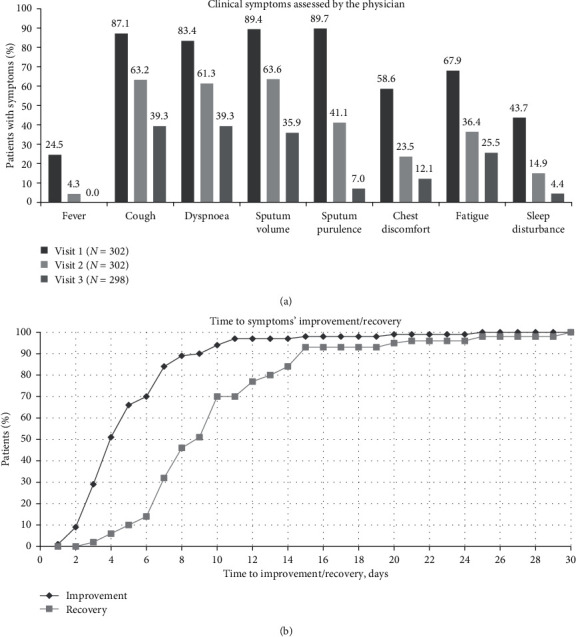
Patient clinical symptoms at baseline (visit 1) and the postbaseline visits and improvement/recovery during the course of the study. (a) Patients with symptoms at baseline, visit 2, and visit 3. (b) Days to the overall improvement and recovery of symptoms.

At baseline, 89.7% (271/302) of the patients had sputum purulence, 89.4% (270/302) increased sputum volume, 87.1% (263/302) cough, and 83.4% (252/302) dyspnoea (Figure 2(a)), which, according to the physician's assessment, were of moderate-to-severe intensity in 76.0% (206/271), 83.3% (225/270), 71.9% (189/263), and 81.0% (204/252) of the patients, respectively. The percentages of patients with these and other AECB symptoms gradually decreased from baseline at visit 2 and visit 3 (Figure 2(a)).

By the end of the study observation period, an improvement in the patients' symptoms was seen in 301 of 302 evaluable patients. The mean (SD) time until improvement was 5.47 (3.57) days (95% CI of the mean: 5.06–5.87). Symptoms' improvement occurred by 4 days in 50.5%, 6 days in 69.8%, 8 days in 89.0%, and 10 days in 93.7% of the patients (Figure 2(b)). Specifically, 91.7% of the patients had improved by visit 2, while of the remaining 8.3%, all but one had improved at visit 3.

Moreover, a recovery in symptoms was reported for 288 patients by the end of the observation period. The mean (SD) time until recovery was 10.22 (5.00) days (95% CI of the mean: 9.64–10.80) (Figure 2(b)). At visit 2, 45.4% of the patients had recovered. Of the 165 patients who had not recovered at visit 2, the evaluation was missing for three cases, while all but 11 of the 162 remaining patients had recovered by visit 3.

**Figure 3:**
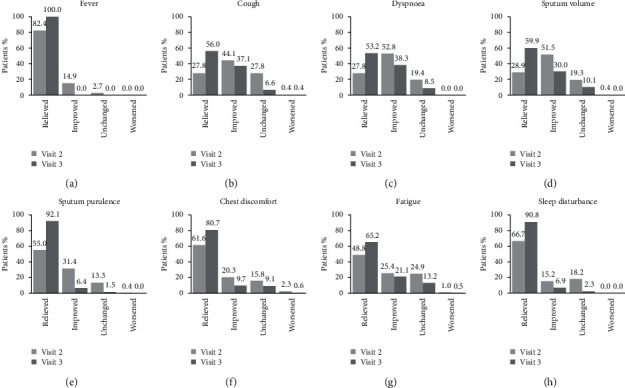
Course of clinical symptoms at visit 2 and visit 3 among patients with symptoms at baseline. Patients with (a) fever, (b) cough, (c) dyspnoea, (d) sputum volume, (e) sputum purulence, (f) chest discomfort, (g) fatigue, and (h) sleep disturbance at baseline whose symptoms were relieved (i.e., switched to absent), improved (decreased intensity), unchanged, or worsened (increased severity) at visit 2 and visit 3.

The distribution of patients with symptoms at baseline according to the course of their clinical symptoms (improved, relieved, unchanged, and worsened) at the postbaseline visits is presented in Figure 3.

### 3.4. Overall Assessment of the Effectiveness of Prulifloxacin

At visits 2 and 3, physicians rated the effectiveness of prulifloxacin as “very good” or “good” in 84.4% (255/302) and 86.9% (259/298) of the evaluable patients, respectively, “sufficient” in 10.3% (31/302) and 8.1% (24/298), and “insufficient” in 5.3% (16/302) and 5.0% (15/298), respectively. In addition, 94.0% (284/302) and 94.3% (281/298) of evaluable patients at visits 2 and 3 reported that prulifloxacin treatment helped their symptoms.

### 3.5. Effects of Prulifloxacin on the Patients' QoL, Work Productivity, and Activity Impairment

**Figure 4:**
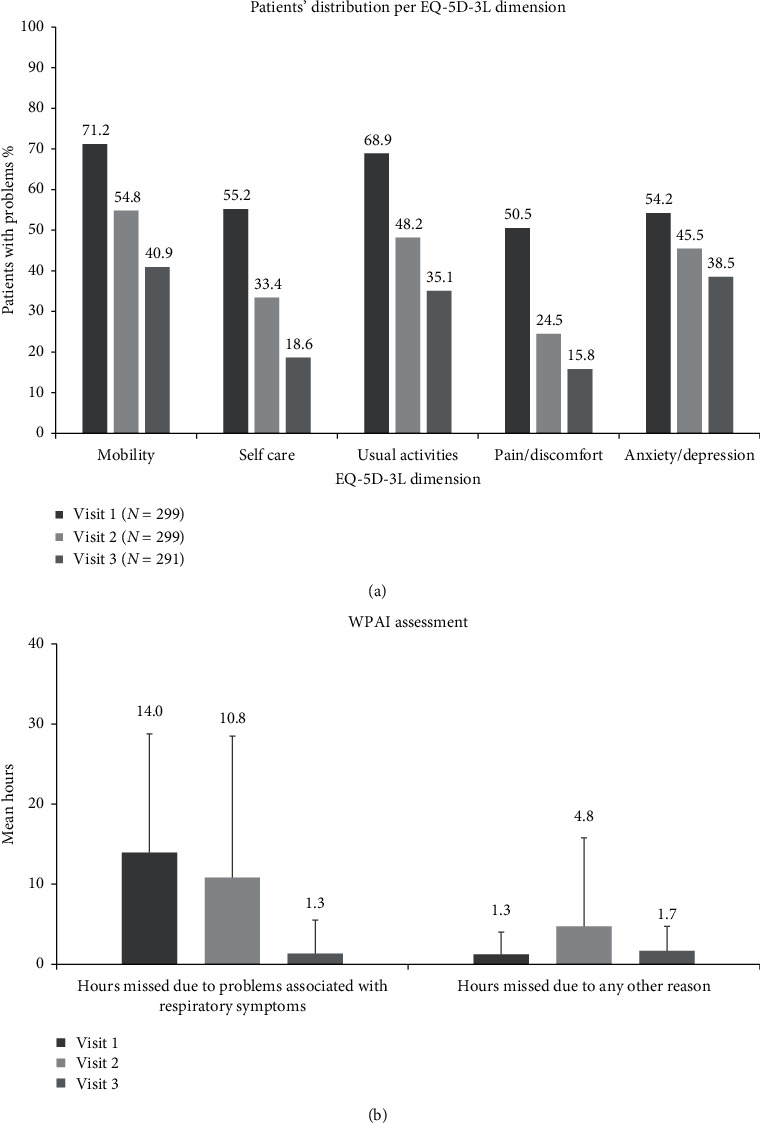
Patients' quality of life and work productivity improvement at baseline (visit 1) and postbaseline visits. (a) Patients with problems in the EQ-5D-3L dimensions mobility, self-care, usual activities, pain/discomfort, and anxiety/depression at baseline, visit 2, and visit 3. (b) Mean hours missed from work due to respiratory and other problems at baseline, visit 2, and visit 3.

The proportion of patients reporting problems in the EQ-5D-3L dimensions at baseline gradually decreased at the postbaseline visits (Figure 4(a)). The EQ-visual analogue scale (EQ-VAS) score improved from a mean (SD) of 55.1 (14.1) points at baseline to 68.3 (12.3) at visit 2 and 75.2 (11.7) at visit 3.

With respect to work status, 9.2% (27/295), 8.8% (26/297), and 8.2% (24/291) of evaluable patients reported being employed at enrolment, visit 2, and visit 3, respectively. Work absenteeism gradually improved at the postbaseline timepoints, as indicated by the reduction of work hours lost (Figure 4(b)). In addition, patients reported an improvement in their work productivity. Specifically, on a scale from 0 to 10, with 0 indicating “no effect” and 10 “severe impairment,” work productivity improved from a mean (SD) of 5.5 (2.9) at baseline to 3.8 (3.5) at visit 2 and 1.6 (1.8) at visit 3. In addition, patients reported an improvement in the extent to which their respiratory symptoms affected their daily activities with the scores on a scale from 0 to 10, decreasing from a mean (SD) of 6.3 (2.1) among 295 evaluable patients at baseline to 4.8 (2.2) and 4.1 (2.8) at visit 2 (*N* = 297) and visit 3 (*N* = 290), respectively.

### 3.6. Safety and Tolerability

The overall tolerability of prulifloxacin was rated by the physicians as “very good”/“good” or “sufficient” in 94.4% (285/302) and 5.0% (15/302) of the patients at visit 2 and in 95.3% (284/298) and 4.0% (12/298) of the patients at visit 3. Tolerability was rated as “insufficient” in two patients (0.7%) at each visit.

A total of 16 AEs were experienced by 4.6% (14/302) of the patients in the safety set, of which four (by four patients; rate: 1.3%) were assessed as related to prulifloxacin (Table 3). Of the latter, two were serious: one event of diarrhoea and a death due to cardiorespiratory arrest (Table 3). The patient who died was an 80-year-old male, who initiated treatment with prulifloxacin for COPD and AECB. The patient attended the outpatient clinic due to symptom exacerbation two days after treatment onset, at which point he was administered corticosteroid and antibiotic treatment for management of the exacerbation. The patient's death occurred on the same day; its causal relationship to the study drug could not be excluded and the event was thus considered “unassessable-unclassifiable.”

## 4. Discussion

The current multicentre, prospective, observational study provides real-world data demonstrating high rates of symptom improvement and resolution in patients treated with prulifloxacin in the routine care setting of Greece, which were accompanied by QoL, work productivity, and activity impairment improvements, high patient and physician satisfaction with treatment, and a good safety and tolerability profile.

The results of this study are consistent with those of RCTs [12–14]. In one RCT, the response rate (clinical cure/improvement) of treatment with prulifloxacin for 10 days was approximately 85% [13]. In addition, a 92.5% treatment success rate was reported in a trial comparing prulifloxacin to amoxicillin/clavulanate [12], while a response rate of 96.7% on Day 10 of prulifloxacin treatment was shown in a sample of patients with COPD suffering an acute exacerbation unresponsive to previous antibiotics [17]. Similarly, the current study demonstrated a 91.7% symptom improvement rate at the 7-10-day postbaseline follow-up visit. This rate was 99.7% at the late follow-up visit (which occurred at least 28 days after treatment completion), at which time 95.4% of the patients had fully recovered. Likewise, in another RCT of patients with confirmed severe COPD whose AECB was managed with prulifloxacin for 7 days, a cure rate of 92.5% was observed at the test of cure visit (7–10 days after treatment discontinuation) [14].

Several baseline characteristics should be taken into consideration when interpreting the study outcomes. About 70% of the patients were ≥65 years of age, approximately 25% had bronchitis duration >10 years, 93.8% patients had FEV1% ≤80, 94.8% had a type I or II exacerbation according to Anthonisen criteria, and 24.6% had cardiac disorders. These comprise some of the factors that have been implicated in lower rates and/or late recovery from an acute exacerbation in previous works [18–21]. In the present study, the mean time to recovery was 10.2 days, which, although higher than that reported in the overall population of the GIANT study assessing the effectiveness of moxifloxacin, is exactly the same as that reported in the study's late recovery group (defined as ≥8 days to recovery), whose baseline characteristics, such as age ≥65 years, bronchitis duration >10 years, and presence of Anthonisen type I/II exacerbation resemble those of the present study more closely than the overall GIANT population [18]. Moreover, the mean time to symptoms' improvement was 5.47 days in the present study, which is longer than that reported in studies with moxifloxacin (mean 3.2–3.4 days), with at least some of the variation likely accounted for by differences in population characteristics as noted above [18, 22, 23]. Furthermore, by the end of treatment, more than 94% of the patients reported being satisfied with their treatment.

Symptom improvement and recovery were accompanied by improvement in the patients' QoL. Specifically, the EQ-VAS score at baseline was 55.1 compared to a mean age-standardized EQ-VAS of 76.5 in the general population of Greece [24]. Notably, this score nearly reached the general population standard at the late follow-up visit (75.2). Alongside the improvement in the EQ-VAS and decreases in the number of patients with problems in all EQ-5D dimensions, the patients reported missing fewer hours from work and an improvement in their activity limitation due to respiratory problems. The link between AECB, and QoL and work productivity impairments has been documented elsewhere [4, 25].

In addition, prulifloxacin demonstrated a manageable safety profile, with the incidence rate of related AEs being 1.3%, similar to that reported in other studies with fluoroquinolones of a similar design and follow-up duration [16, 22, 23]. The observed events are among those expected based on accumulated safety data for prulifloxacin and the class of fluoroquinolones [17, 26]. Physicians rated the tolerability of prulifloxacin as “very good” or “good” in 94.4% of the patients.

Collectively, the above information demonstrates the effectiveness of prulifloxacin in the management of patients with moderate-to-severe AECB, including elderly patients with a severe decline in lung function. Combined with prulifloxacin's wide spectrum activity against the most common pathogens associated with AECB, its good penetration in lung tissues, and its long half-life, which allows for a once daily administration and, thus, increased patient compliance [12, 20], the study results support its use for the management of AECB in cases indicated under the approved label.

The results of AIOLOS complement prior studies on the use of antibiotics in AECB. With guidelines on the management of exacerbations of COPD and chronic bronchitis being regularly updated, antibiotics remain a valid option in the treatment algorithm; however, optimal antibiotic selection decision should be based on careful patient-centered risk/benefit assessment of the available options [27]. Some classes display a better safety and tolerance profile, others are superior in terms of bacterial eradication, immunomodulation, and control of inflammation or achieve longer exacerbation-free intervals, and another concern is the development of resistance [28–34]. For instance, comparison between the fluoroquinolone levofloxacin and the macrolide clarithromycin showed that the two drugs had similar clinical success rates, with no significant differences in the exacerbation-free intervals, but treatment with levofloxacin was associated with a higher bacteriological success rate [28]. Similar treatment failure rates were also observed upon comparison of macrolides with quinolones, although the former was associated with lower incidence of diarrhoea [29]. Moreover, macrolides possess anti-inflammatory and immunomodulatory actions which are of relevance to the treatment of COPD [31, 32]. The difference in resistance-selection properties among specific antibiotics is another confounding factor, as in vitro data support that they can vary even among agents of the same class [33, 34]. The physician is called to make the optimal decision, taking into account the aforementioned parameters, as well as the patient and disease characteristics on a case-by-case basis.

The present study is primarily limited by the lack of a control group, which does not allow for direct inferences as to whether the improvement of patients' symptoms is attributable to the effect of the antibiotic or depicts the natural course of the exacerbation. Other limitations include the absence of data on microbiological examinations, as well as the fact that physicians' assessments, though accurately depicting routine clinical practice, may be subjective, as no validated tools have been employed. On the other hand, with respect to the assessments performed using the WPAI : RS and EQ-5D-3L instruments, little or no recall bias is expected, as both tools employ a short recollection period ranging from 0 to 7 days. As all patients were enrolled by pulmonology specialists practicing in hospital outpatient clinics, the study results are generalizable to outpatients managed by hospital-based physicians, rather than patients cared for in all ambulatory care settings including private practices. On the other hand, as the study was designed to target patients with moderate-to-severe exacerbations, it is inherently more likely that such patients proceed to hospital settings to receive the necessary care. Moreover, enrolment of the patients by 13 public and private hospital clinics from geographically diverse locations of Greece where 80% of the country's population resides accounts for variations in medical practice and strengthens the generalizability of the outcomes. Notably, the study had a very low attrition rate, while in fact all four patients of the effectiveness population who did not attend the late follow-up visit had achieved complete resolution of their symptoms at visit 2, thus preventing bias arising from selectively missing data from patients who prematurely discontinued study participation due to inadequate response. Lastly, three patients had used antibiotics in the month prior to enrolment and four received antibiotics concomitantly to prulifloxacin, a fact that may have increased the rates of the observed effectiveness outcomes, albeit to a small extent given the small number of such cases. It should further be noted that as this study was conducted in the routine care, all decisions of the physicians regarding length of treatment with prulifloxacin and concomitant medications were based solely on the physicians' medical judgment and, therefore, better reflect real-world practice.

## 5. Conclusions

In conclusion, this real-world study demonstrates the effectiveness of prulifloxacin in the treatment of moderate-to-severe AECB. Symptoms' improvement was noted within 6 days in seven out of 10 patients, while by the end of the study a complete resolution of symptoms occurred in more than nine out of 10 cases. These findings were accompanied by improvements in the patients' QoL, work productivity, and activity limitation, a predictable safety profile, and high level of patient satisfaction with therapy.

## Figures and Tables

**Table 1:** Patient and disease characteristics at baseline.

Patient baseline characteristics	
Males (*N* = 305), *n* (%)				233 (76.4)
Caucasian (*N* = 305), *n* (%)				305 (100.0)
Age (*N* = 305), mean (SD), years				69.7 (9.8)
≥65 years old (*N* = 305), *n* (%)				217 (71.1)

*Smoking status (N* *=* *305), n (%)*		
Ex‐smokers				204 (66.9)
Current smokers				85 (27.9)
At least one past or ongoing medical condition/surgery/comorbidity (*N* = 305), *n* (%)				220 (72.1)
At least one ongoing medical condition/comorbidity (*N* = 305), *n* (%)				208 (68.2)
*Past or ongoing medical conditions/comorbidities in ≥10% of the patients (N* *=* *305), n (%)*				
Essential hypertension				109 (35.7)
Cardiac disorders				75 (24.6)
Dyslipidaemia				47 (15.4)
Benign prostatic hyperplasia				36 (11.8)
Diabetes mellitus				37 (12.1)

*Disease characteristics at baseline*		
Duration of chronic bronchitis (*N* = 305), mean (SD), years				7.96 (6.22)
≤5 years				149 (48.9)
>5 to ≤10 years				80 (26.2)
>10 years				76 (24.9)

*Classification according to Anthonisen criteria (N* *=* *305), n (%)*		
Type I				156 (51.1)
Type II				133 (43.6)
Type III				16 (5.2)

FEV1% (*N* = 305), mean (SD)				52.90 (15.35)
≤50				126 (41.3)
>50 to ≤80				160 (52.5)
>80				19 (6.2)

*Number of exacerbations in the previous year (N* *=* *305), n (%)*		
0				68 (22.3)
1				154 (50.5)
2				42 (13.8)
3				26 (8.5)
≥4				15 (4.9)
Patient visits to physicians for exacerbations in the previous year (*N* = 305), *n* (%)				223 (73.1)
Number of visits to physicians for exacerbations in the past 12 months (*N* = 223), mean (SD)				1.72 (1.32)
Patients hospitalized for exacerbations in the past 12 months (*N* = 305), *n* (%)				45 (14.8)
Number of hospitalizations for exacerbations in the past 12 months (*N* = 45), mean (SD)				1.4 (0.9)
Length of hospitalizations for exacerbations in the past 12 months (*N* = 45), mean (SD)				9.0 (5.5)

FEV1: forced expiratory volume in 1 second; SD: standard deviation.

**Table 2:** Prulifloxacin treatment characteristics.

Prulifloxacin treatment characteristics	
Prulifloxacin administration at the recommended dose of 600 mg once daily (*N* = 302), *n* (%)	297 (98.3)
Prulifloxacin treatment duration (*N* = 298), mean (SD), days	9.53 (1.28)
Patients' distribution per treatment duration in days (*N* = 302), *n* (%)	
≤6	11 (3.6%)
7	23 (7.6%)
8	6 (2.0%)
9	7 (2.3%)
10	255 (84.4%)
Prulifloxacin tablets taken (*N* = 302), mean (SD)	9.53 (1.28)

SD: standard deviation.

**Table 3:** Incidence of adverse events.

Adverse events, *N* = 305	Events	*n* (%)
*At least one adverse event*	16	14 (4.6)
Chronic obstructive pulmonary disease	3	3 (1.0)
Diarrhoea	2	2 (0.7)
Pneumonia	2	2 (0.7)
Bronchitis	1	1 (0.3)
Condition aggravated	1	1 (0.3)
Death	1	1 (0.3)
Fatigue	1	1 (0.3)
Gastrointestinal disorder	1	1 (0.3)
Headache	1	1 (0.3)
Nausea	1	1 (0.3)
Vomiting	1	1 (0.3)
Plasma cell myeloma	1	1 (0.3)

*At least one serious adverse event*	7	6 (2.0)
Chronic obstructive pulmonary disease^a^	2	2 (0.7)
Pneumonia^a^	2	2 (0.7)
Plasma cell myeloma^a^	1	1 (0.3)
Diarrhoea	1	1 (0.3)
Death	1	1 (0.3)

At least one adverse event assessed to be related to prulifloxacin	4	4 (1.3)
At least one *serious* adverse event assessed to be related to prulifloxacin	2	2 (0.7)
Diarrhoea	1	1 (0.3)
Death	1	1 (0.3)

At least one *nonserious* adverse event assessed to be related to prulifloxacin	2	2 (0.7)
Diarrhoea	1	1 (0.3)
Headache	1	1 (0.3)

^a^The four patients who experienced these events were hospitalized. One patient experienced two serious adverse events, namely, pneumonia and plasma cell myeloma.

## Data Availability

The datasets generated and analysed during the current study are not publicly available due to concerns regarding the possibility for individual patients to be identified but are available from the corresponding author upon reasonable request.

## Supplementary Materials

Supplementary Materials STROBE statement: checklist of items that should be included in reports of cohort studies.

## References

[1] Albertson T. E., Louie S., Chan A. L. (2010). The diagnosis and treatment of elderly patients with acute exacerbation of chronic obstructive pulmonary disease and chronic bronchitis. *Journal of the American Geriatrics Society*.

[2] Blasi F., Ewig S., Torres A., Huchon G. (2006). A review of guidelines for antibacterial use in acute exacerbations of chronic bronchitis. *Pulmonary Pharmacology & Therapeutics*.

[3] Doll H., Grey-Amante P., Duprat-Lomon I. (2002). Quality of life in acute exacerbation of chronic bronchitis: results from a German population study. *Respiratory Medicine*.

[4] Schmier J. K., Halpern M. T., Higashi M. K., Bakst A. (2005). The quality of life impact of acute exacerbations of chronic bronchitis (AECB): a literature review. *Quality of Life Research*.

[5] Hillas G., Perlikos F., Tzanakis N. (2016). Acute exacerbation of COPD: is it the “stroke of the lungs”?. *International Journal of Chronic Obstructive Pulmonary Disease*.

[6] Minas M., Verrou-Katsarou I., Mystridou P., Apostolidou E., Hatzoglou C., Gourgoulianis K. I. (2012). Two-year mortality of patients with COPD in primary health care: an observational study. *International Journal of General Medicine*.

[7] Anthonisen N. R., Manfreda J., Warren C. P., Hershfield E. S., Harding G. K., Nelson N. A. (1987). Antibiotic therapy in exacerbations of chronic obstructive pulmonary disease. *Annals of Internal Medicine*.

[8] Canut A., Martín-Herrero J. E., Labora A., Maortua H. (2007). What are the most appropriate antibiotics for the treatment of acute exacerbation of chronic obstructive pulmonary disease? A therapeutic outcomes model. *Journal of Antimicrobial Chemotherapy*.

[9] Siempos I. I., Dimopoulos G., Korbila I. P., Manta K., Falagas M. E. (2007). Macrolides, quinolones and amoxicillin/clavulanate for chronic bronchitis: a meta-analysis. *European Respiratory Journal*.

[10] European Medicines Agency (February 2019). Quinolone- and fluoroquinolone-containing medicinal products. https://www.ema.europa.eu/en/medicines/human/referrals/quinolone-fluoroquinolone-containing-medicinal-products.

[11] Lode H., Allewelt M. (2002). Role of newer fluoroquinolones in lower respiratory tract infections. *Journal of Antimicrobial Chemotherapy*.

[12] Cazzola M., Salvatori E., Dionisio P., Allegra L. (2006). Prulifloxacin: a new fluoroquinolone for the treatment of acute exacerbation of chronic bronchitis. *Pulmonary Pharmacology & Therapeutics*.

[13] Grassi C., Salvatori E., Rosignoli M. T., Dionisio P. (2002). Randomized, double-blind study of prulifloxacin versus ciprofloxacin in patients with acute exacerbations of chronic bronchitis. *Respiration*.

[14] Blasi F., Schaberg T., Centanni S., Del Vecchio A., Rosignoli M. T., Dionisio P. (2013). Prulifloxacin versus levofloxacin in the treatment of severe COPD patients with acute exacerbations of chronic bronchitis. *Pulmonary Pharmacology & Therapeutics*.

[15] National Organization for Medicines (2006). *Prixina Summary of Product Characteristics*.

[16] Miravitlles M., Anzueto A., Ewig S., Legnani D., Stauch K. (2009). Characterisation of exacerbations of chronic bronchitis and COPD in Europe: the GIANT study. *Therapeutic Advances in Respiratory Disease*.

[17] Giusti M., Blasi F., Iori I. (2016). Prulifloxacin vs levofloxacin for exacerbation of COPD after failure of other antibiotics. *COPD*.

[18] Anzueto A., Miravitlles M., Ewig S., Legnani D., Heldner S., Stauch K. (2012). Identifying patients at risk of late recovery (≥8 days) from acute exacerbation of chronic bronchitis and COPD. *Respiratory Medicine*.

[19] Wilson R., Jones P., Schaberg T., Arvis P., Duprat-Lomon I., Sagnier P. P. (2006). Antibiotic treatment and factors influencing short and long term outcomes of acute exacerbations of chronic bronchitis. *Thorax*.

[20] Blasi F., Aliberti S., Tarsia P., Santus P., Centanni S., Allegra L. (2007). Prulifloxacin: a brief review of its potential in the treatment of acute exacerbation of chronic bronchitis. *International Journal of COPD*.

[21] Alexopoulos E. C., Malli F., Mitsiki E., Bania E. G., Varounis C., Gourgoulianis K. I. (2015). Frequency and risk factors of COPD exacerbations and hospitalizations: a nationwide study in Greece (Greek obstructive lung disease epidemiology and health economics: GOLDEN study). *International Journal of Chronic Obstructive Pulmonary Disease*.

[22] Barth J., Landen H. (2003). Efficacy and tolerability of moxifloxacin in 2338 patients with acute exacerbation of chronic bronchitis. *Clinical Drug Investigation*.

[23] Chuchalin A., Zakharova M., Dokic D., Tokić M., Marschall H. P., Petri T. (2013). Efficacy and safety of moxifloxacin in acute exacerbations of chronic bronchitis: a prospective, multicenter, observational study (AVANTI). *BMC Pulmonary Medicine*.

[24] Szende A., Janssen B., Cabasés J. (2014). *Self-Reported Population Health: An International Perspective Based on EQ-5D*.

[25] Solem C., Sun S., Sudharshan L., Macahilig C., Katyal M., Gao X. (2013). Exacerbation-related impairment of quality of life and work productivity in severe and very severe chronic obstructive pulmonary disease. *International Journal of Chronic Obstructive Pulmonary Disease*.

[26] Bertino J., Fish D. (2000). The safety profile of the fluoroquinolones. *Clinical Therapeutics*.

[27] Global Initiative for Chronic Obstructive Lung Disease (2021). Global strategy for the diagnosis, management, and prevention of chronic obstructive pulmonary disease. https://goldcopd.org/wp-content/uploads/2020/11/GOLD-REPORT-2021-v1.1-25Nov20_WMV.pdf.

[28] Lode H., Eller J., Linnhoff A., Ioanas M. (2004). Levofloxacin versus clarithromycin in COPD exacerbation: focus on exacerbation-free interval. *European Respiratory Journal*.

[29] Rothberg M. B., Pekow P. S., Lahti M., Brody O., Skiest D. J., Lindenauer P. K. (2010). Comparative effectiveness of macrolides and quinolones for patients hospitalized with acute exacerbations of chronic obstructive pulmonary disease (AECOPD). *Journal of Hospital Medicine*.

[30] Salkind A. R., Cuddy P. G., Foxworth J. W. (2002). Fluoroquinolone treatment of community-acquired pneumonia: a meta-analysis. *Annals of Pharmacotherapy*.

[31] Zarogoulidis P., Papanas N., Kioumis I., Chatzaki E., Maltezos E., Zarogoulidis K. (2012). Macrolides: from in vitro anti-inflammatory and immunomodulatory properties to clinical practice in respiratory diseases. *European Journal of Clinical Pharmacology*.

[32] Huckle A. W., Fairclough L. C., Todd I. (2018). Prophylactic antibiotic use in COPD and the potential anti-inflammatory activities of antibiotics. *Respiratory Care*.

[33] De Vecchi E., Nicola L., Ossola F., Drago L. (2009). In vitro selection of resistance in Streptococcus pneumoniae at in vivo fluoroquinolone concentrations. *Journal of Antimicrobial Chemotherapy*.

[34] Drago L., Nicola L., Mattina R., De Vecchi E. (2010). In vitro selection of resistance in *Escherichia coli* and Klebsiella spp. at in vivo fluoroquinolone concentrations. *BMC Microbiology*.

